# GLP-1 Receptor Agonist Exenatide Protects Against Doxorubicin-Induced Cardiotoxicity Through the SIRT1 Pathway: An Electrocardiographic, 99mTc-PYP Scintigraphic, and Biochemical Study

**DOI:** 10.3390/medicina62010143

**Published:** 2026-01-10

**Authors:** Musa Salmanoglu, Gulcin Ercan, Hanife Seyda Genç, Serdar Savaş Gül, Hatice Aygün

**Affiliations:** 1 Department of Internal Medicine, Sultan 2. Abdulhamid Han Educational and Research Hospital, University of Health Sciences, 34668 Istanbul, Turkey; drmusa1973@gmail.com; 2 Department of General Surgery, Sultan 2. Abdulhamid Han Educational and Research Hospital, University of Health Sciences, 34668 Istanbul, Turkey; ghepgul@hotmail.com (G.E.); hanifeseydaulgur@gmail.com (H.S.G.); 3 Department of Nuclear Medicine, Faculty of Medicine, Lokman Hekim University, 06510 Ankara, Turkey; serdar.gul@lokmanhekim.edu.tr; 4 Neuroscience Laboratory, BAMER, Biruni University, 34015 Istanbul, Turkey

**Keywords:** GLP-1, doxorubicin, cardiotoxicity, exenatide, scintigraphy, ECG

## Abstract

*Background and Objectives:* This study was designed to evaluate the potential cardioprotective effect of Exenatide against doxorubicin (DOX)-induced myocardial injury in rats by assessing scintigraphic alterations together with oxidative stress and inflammation. *Materials and Methods:* This study included 28 adult male Wistar albino rats that were randomized to 4 groups (*n* = 7): control, Exenatide alone, DOX (receiving DOX (18 mg/kg, i.p) on days 5–7; Exenatide + DOX (treated with Exenatide together with the DOX). On day 8, ECG, 99mTc-PYP scintigraphy, and biochemical parameters were evaluated. *Results:* DOX caused ECG abnormalities—bradycardia, significant QT prolongation, and elevated ST-segment amplitude—along with increased myocardial PYP uptake. Exenatide + DOX group significantly improved ECG changes. Biochemically, DOX markedly increased cardiac injury biomarkers (cTnT, CK, CK-MB), hepatic and renal injury markers (ALT, AST, LDH, BUN, creatinine), SIRT-1 level, inflammatory marker (NF-κB, TNF-α, IL-6, NO) and oxidative stress indicators (MDA, TOS), while decreasing antioxidant defenses (GSH, TAS, Nrf2). Exenatide co-treatment significantly attenuated all DOX-induced changes. *Conclusions:* Exenatide markedly attenuates DOX-induced cardiotoxicity by improving electrical conduction, reducing myocardial radiotracer uptake, and restoring oxidative–inflammatory balance through partial recovery of the SIRT-1/Nrf2/NF-κB pathway.

## 1. Introduction

Doxorubicin (DOX) is an effective anthracycline chemotherapeutic, yet its clinical use is constrained by cumulative dose–dependent cardiotoxicity [1]. Clinically, DOX-induced cardiotoxicity encompasses a broad spectrum, ranging from early pericarditis–myocarditis syndrome and acute ventricular dysfunction to late-onset dilated cardiomyopathy [2]. Although oxidative stress, mitochondrial dysfunction, apoptosis, and inflammation all contribute to DOX cardiotoxicity, none fully explain its multifactorial pathology, underscoring the need for improved cardioprotective strategies.

NF-κB is a central mediator of DOX-induced cytokine activation [3]. SIRT-1, a class III histone deacetylase involved in apoptosis and cell-cycle control, plays a critical role in myocardial development and cardiac homeostasis, and its dysregulation has been associated with structural and functional cardiac abnormalities [4,5]. Importantly, SIRT-1 inhibits NF-κB transcriptional activity [5], suggesting a key upstream role in modulating DOX-related inflammatory injury.

Exenatide, a GLP-1 receptor agonist used in type 2 diabetes, has demonstrated antioxidant and anti-inflammatory properties, along with cardioprotective effects in experimental and clinical settings [6,7]. Experimental studies show that GLP-1R activation improves ischemic tolerance and ventricular function through pro-survival kinase pathways [8]. Exenatide reduces ROS generation, enhances antioxidant enzymes, limits mitochondrial calcium overload, and stabilizes membrane potential [9]. It also suppresses myocardial TLR-4/NF-κB signaling and elevates SIRT-1, thereby reducing oxidative and inflammatory myocardial injury [10].

Although SIRT-1 and Exenatide independently modulate oxidative and inflammatory responses, the role of SIRT-1 in Exenatide-mediated protection during acute DOX cardiotoxicity remains unclear. Therefore, this study aimed to determine whether SIRT-1 contributes to Exenatide’s cardioprotective effects in a DOX-induced cardiotoxicity model, using 99mTc-PYP scintigraphy, ECG monitoring, and comprehensive biochemical analyses.

## 2. Materials and Methods

### 2.1. Animal

Male Wistar Albino rats weighing approximately 230–250 g were used. Prior to experimental procedures, all animals were allowed a one-week acclimatization period in the laboratory, during which they had unrestricted access to food and water. All interventions were performed between 09:00 and 10:00 each day to preserve the animals’ diurnal rhythm. The rats were housed in standard cages under controlled environmental conditions, including a room temperature of 22–25 °C and a 12 h light/12 h dark cycle. The animals were obtained from the Tokat Gaziosmanpaşa University Laboratory Animal Facility. All procedures were carried out in accordance with the ARRIVE guidelines. Ethical approval for the study was granted by the Tokat Gaziosmanpasa University Local Animal Experiments Ethics Committee (2017 HADYEK-20).

### 2.2. Experimental Design

**Figure 1:**
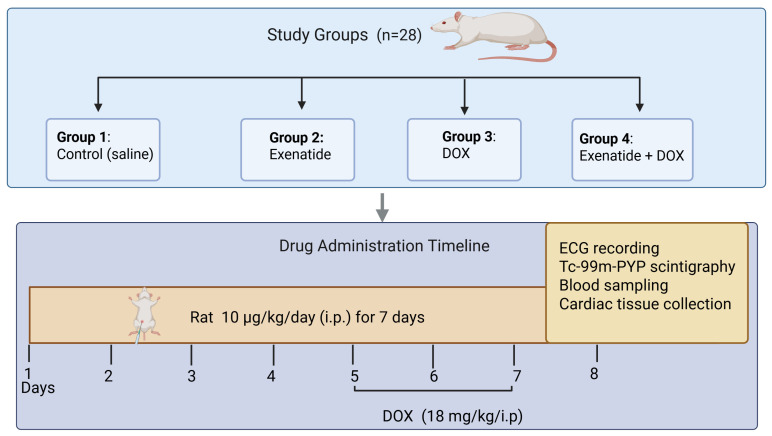
Overview of the experimental groups, treatment protocol, and procedural timeline. DOX, and Exenatide + DOX. Exenatide was administered intraperitoneally at 10 µg/kg/day for seven consecutive days, while a single dose of DOX was administered on days 5–7 (cumulative dose: 18 mg/kg, i.p.). On day 8, all animals underwent electrocardiographic (ECG) assessment followed by 99mTc-PYP scintigraphic imaging. Subsequently, blood samples were collected for biochemical analysis, and cardiac tissues were harvested for molecular and histological evaluation.

The rats were randomly assigned to three experimental groups. The control animals received only intraperitoneal saline throughout the study. In the DOX group, the drug was administered intraperitoneally (i.p.) on experimental days 5, 6, and 7, yielding a cumulative dose of 18 mg/kg. The third group was treated with exenatide at 10 μg/kg/day for seven consecutive days, administered each morning at 09:00. These animals also received doxorubicin on days 5–7 following the same cumulative dosing scheme as the DOX-only group [11] (Figure 1).

Group I—Control: Rats received no pharmacological treatment other than intraperitoneal saline.

Group II—Exenatide: Rats received daily intraperitoneal injections of Exenatide at a dose of 10 µg/kg/day (i.p.) at 09:00 for seven consecutive days.

Group III—Doxorubicin: Rats were administered DOX on days 5, 6, and 7 of the experiment (cumulative dose: 18 mg/kg, intraperitoneally).

Group IV—Exenatide + Doxorubicin: Animals in this group received Exenatide at a dose of 10 μg/kg/day (i.p.) for seven consecutive days at 09:00 each morning. In addition, DOX was administered on days 5, 6, and 7 (cumulative dose: 18 mg/kg, i.p.), following the same schedule as Group III.

On day 8, ECG recordings were obtained from all animals first. This was followed by scintigraphic imaging. After completion of the scintigraphy procedure, blood samples were collected via cardiac puncture under deep anesthesia for biochemical analyses. Finally, the animals were euthanized by cervical dislocation under enhanced anesthesia. The heart was rapidly excised, rinsed in cold saline, and stored at −80 °C until biochemical and molecular assays were conducted. The overall study workflow is summarized below.

### 2.3. Drug

In accordance with earlier studies showing that a daily 10 µg/kg Exenatide regimen produces reliable metabolic, neuroprotective, and anti-inflammatory effects in rodent disease models, the same dose was adopted in the present experiment [12,13]. The DOX doses were determined according to previous studies [11].

### 2.4. Electrocardiography (ECG) Procedure

**Figure 2:**
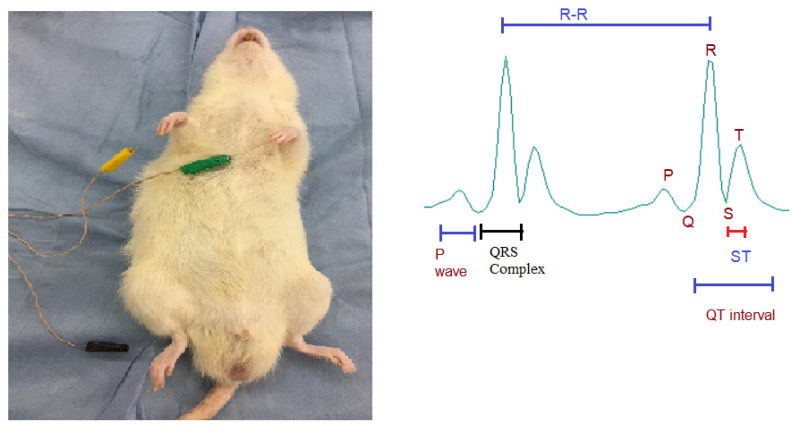
Lead II ECG recording setup and representative waveform components. Placement of needle electrodes for Lead II ECG acquisition in anesthetized rats (**left**), and schematic illustration of major ECG intervals including P wave, QRS complex, ST segment, QT interval, and R–R interval (**right**).

A standard Lead II ECG was recorded on day 8 under ketamine (75 mg/kg) and xylazine (10 mg/kg) anesthesia. Depth of anesthesia was assessed clinically by the pedal withdrawal reflex. Subcutaneous needle electrodes were placed on both forelimbs and the left hindlimb. After a stable baseline, a 60 s tracing was acquired using the BIOPAC MP150 system, and ECG intervals were digitally quantified. Representative waveform components (P wave, QRS complex, QT interval, ST segment) are illustrated in Figure 2. All recordings were performed under stable anesthetic depth to reduce respiratory and motion artifacts.

### 2.5. Scintigraphic Imaging Procedure

After ECG acquisition, scintigraphic imaging was performed. For this purpose, 1 mCi of 99m Tc-PYP radiopharmaceutical (TechneScan PYP, Mallinckrodt, Québec, CA, Canada) was diluted in 5 mL of isotonic saline, and 0.1 mL of this preparation was administered intraperitoneally to each rat. One hour post-injection, static images were acquired with a gamma camera (Siemens Symbia, Erlangen, Germany). Regions of interest (ROIs) were manually drawn over the affected myocardial areas, and seven separate measurements were obtained for each animal; mean ROI values were then calculated and used for quantitative analysis.

### 2.6. Biochemical Assays

For the biochemical evaluations, roughly 5 mL of blood was withdrawn from the abdominal aorta and transferred into serum-separator Vacutainer tubes (gold-capped). Serum samples were allowed to clot for 30 min, centrifuged at 1500× *g* for 10 min, and the clear supernatant was collected for biochemical analysis.

### 2.7. Quantification of Cardiac, Renal, and Hepatic Biomarkers

Cardiac injury markers were quantified using commercially available kinetic assay kits. Serum levels of lactate dehydrogenase (LDH), aspartate aminotransferase (AST), alanine aminotransferase (ALT), creatine kinase (CK), its myocardial isoform CK-MB, blood urea nitrogen (BUN), creatinine, and cardiac troponin-T (cTnT) were quantified by kinetic spectrophotometric methods using commercially available kits on the Beckman Coulter LX-2000 analyzer (Beckman Coulter, Inc., Brea, CA, USA).

### 2.8. Biochemical Analysis of Heart Tissue

All tissue samples were rinsed with ice-cold isotonic saline (0.9%) and weighed to obtain wet tissue mass. The specimens were then minced into small fragments using a scalpel and transferred into microtubes containing stainless-steel beads. Homogenization was performed in cold PBS (pH 7.4). The homogenates were centrifuged at 2500× *g* for 20 min, and the resulting supernatants were collected for biochemical assays.

Tissue concentrations of cardiac tissue homogenates were evaluated using ELISA to quantify SIRT-1, NF-κB, and Nrf2 levels, and tissue GSH, total antioxidant status (TAS), and total oxidant status (TOS) were quantified using commercially available quantitative sandwich ELISA kits, following the manufacturer’s instructions. All results were normalized to wet tissue weight and expressed as ng/mg for SIRT-1, µmol/L/g for TOS and GSH, mmol/L for TAS, (pg/mg) for NF-κB.

### 2.9. ELISA Kit Information

Plasma and cardiac tissue SIRT-1 levels were quantified using a rat-specific ELISA kit supplied by Elabscience (Houston, TX, USA; Elabscience^®^, Cat. No: E-EL-R1102). Cardiac NF-κB p65 protein concentrations were determined with a commercial ELISA kit from Abcam (Abcam, Cambridge, UK; Cat. No: ab176648). Nrf2 levels were measured using the Rat Nrf2 ELISA kit obtained from MyBioSource (MyBioSource, Cat. No: MBS012148, Inc., San Diego, CA, USA). Plasma malondialdehyde (MDA) concentrations were assessed with a rat-specific MDA ELISA kit provided by MyBioSource (MyBioSource, San Diego, CA, USA, Cat. No: MBS268427). Glutathione (GSH) levels were analyzed using a colorimetric assay kit from Invitrogen/Thermo Fisher Scientific (Invitrogen™, Cat. No: EEA020). IL-6 levels were quantified with the Rat Interleukin-6 ELISA kit (Waltham, MA, USA; Catalog No: E0135Ra), Nitric oxide (NO) concentrations were determined using the Rat Nitric Oxide ELISA kit (Houston, TX, USA; Catalog No: E0703Ra), TNF-α was analyzed using the corresponding rat-specific ELISA kit (E0764Ra), and all procedures were carried out strictly according to the supplier’s instructions. Calibration standards provided within each kit were used to generate standard curves, and final concentrations were normalized to tissue weight prior to statistical analysis.

### 2.10. Statistical Analysis

Before performing group comparisons, the distribution of each dataset was evaluated using the Shapiro–Wilk normality test. For variables that met the assumption of normal distribution, one-way ANOVA was applied, followed by Tukey’s (or Bonferroni’s, where appropriate) post hoc multiple comparison test. Homogeneity of variances was assessed, and when variance equality was violated, Welch-corrected ANOVA and Tamhane’s post hoc test were used. For parameters that did not follow a normal distribution, non-parametric analyses were performed using the Mann–Whitney U test for pairwise comparisons or the Kruskal–Wallis test for multiple groups. A *p*-value of <0.05 was considered statistically significant. Graphs were generated using GraphPad Prism 9, and all statistical analyses were performed using SPSS version 19. All data were expressed as mean ± SEM.

## 3. Results

### 3.1. ECG Findings

ECG tracings were evaluated for heart rate, QT interval, and ST-segment elevation (Table 1). One-way ANOVA revealed a significant group effect of Heart rate (F(3,24) = 12.644, *p* < 0.001), QT interval (F(3,24) = 23.100, *p* < 0.001), and ST segment (F(3,24) = 39.289, *p* < 0.001). DOX administration significantly reduced heart rate compared with the Control group (*p* < 0.001). Pretreatment with Exenatide partially increased the heart rate compared with the DOX group (*p* = 0.032 vs. DOX). DOX administration caused a marked prolongation of the QT interval compared with Control (*p* < 0.001). Exenatide co-treatment significantly attenuated DOX-induced QT prolongation when compared with the DOX group (*p* < 0.01). ST elevation was significantly increased in DOX-treated rats (*p* < 0.001). ST elevation induced by DOX is significantly reversed by Exenatide treatment (*p* < 0.01).

The differences in heart rate, QT interval, and ST segment elevation between the Exenatide treatment alone and the control group were not statistically significant (*p* > 0.05). The Exenatide + DOX group still exhibited a significantly prolonged QT interval and increased ST-segment amplitude compared with the Control group (*p* < 0.05, *p* < 0.01, respectively). In contrast, heart rate did not differ between the groups.

**Figure 3:**
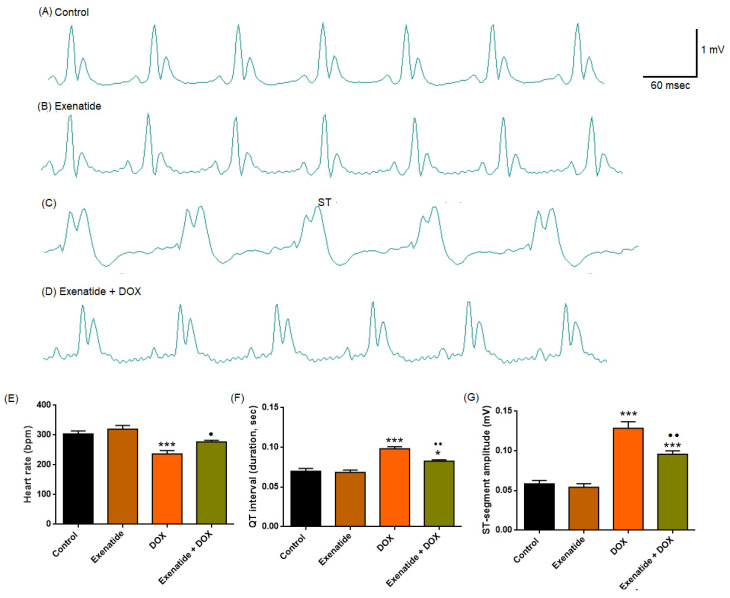
Effects of Exenatide on DOX-induced alterations in electrocardiographic parameters. (**A**) Representative ECG recordings from the control group demonstrate normal P–QRS–T wave morphology. (**B**) In the exenatide-treated group, the ECG pattern is preserved and comparable to that of the control group. (**C**) The DOX group exhibits marked ST-segment elevation and prolongation of the QT interval. (**D**) In the exenatide + DOX group, DOX-induced ECG abnormalities are markedly attenuated. (**E**) Heart rate (bpm), (**F**) QT interval duration (s), and (**G**) ST-segment amplitude (mV) in Control, Exenatide, DOX, and Exenatide + DOX groups. Exenatide co-administration partially restored these DOX-induced electrophysiological abnormalities, as reflected by improved heart rate, QT interval, and attenuated ST elevation. Data are presented as mean ± SEM (*n* = 7 per group). * *p* < 0.05, *** *p* < 0.001 vs. Control. • *p* < 0.05, •• *p* < 0.01, vs. DOX. Scale bars: 1 mV (vertical) and 60 ms (horizontal).

DOX administration resulted in characteristic cardiotoxic electrical changes, including reduced heart rate, pronounced QT prolongation, and elevated ST-segment amplitude. Exenatide alone exhibited no adverse electrophysiological effects and significantly mitigated DOX-induced disturbances across all major parameters, demonstrating a clear cardioprotective profile (Figure 3).

### 3.2. Serum Analysis

#### 3.2.1. Myocardial Injury Biomarkers

**Figure 4:**
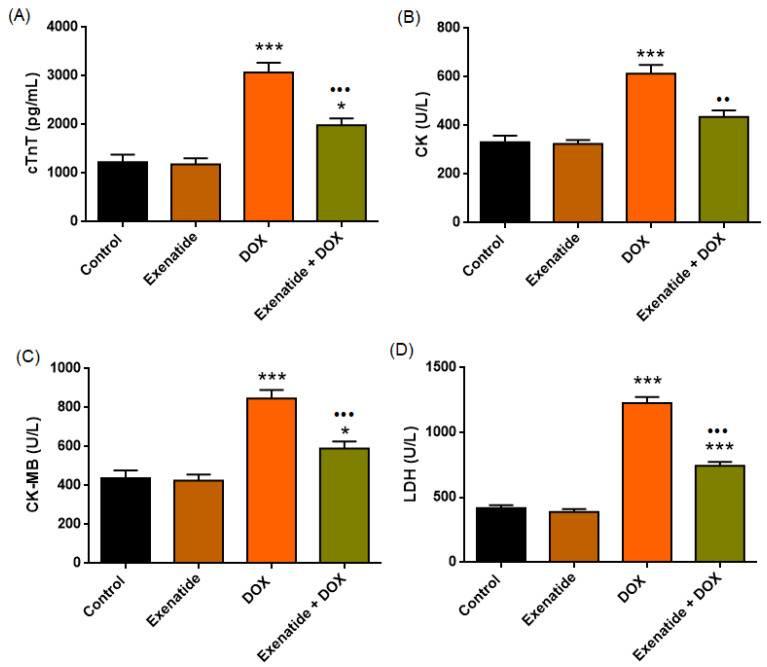
Effects of Exenatide on DOX-induced myocardial injury biomarkers. (**A**) Plasma cTnT (pg/mL), (**B**) serum CK (U/L), (**C**) serum CK-MB (U/L), (**D**) lactate dehydrogenase (LDH, U/L) levels in Control, Exenatide, DOX, and Exenatide + DOX groups. DOX administration markedly increased all three myocardial injury biomarkers, indicating severe cardiomyocyte damage. Exenatide alone showed no effect, whereas Exenatide co-treatment significantly attenuated DOX-induced elevations, although partial elevations persisted compared with Control. * *p* < 0.05, *** *p* < 0.001 vs. Control. •• *p* < 0.01, ••• *p* < 0.001 vs. DOX.

The distribution of serum myocardial injury biomarkers cTnT, CK, and CK-MB in the four groups is as shown in Table 2. One-way ANOVA revealed highly significant treatment effects on circulating cTnT (F(3,24) = 32.166, *p* < 0.001), CK (F(3,24) = 23.908, *p* < 0.001), CK-MB levels (F(3,24) = 26.760, *p* < 0.001) and LDH (F(3,24) = 146.200, *p* < 0.001). In all biomarkers, doxorubicin produced a pronounced elevation compared with the Control and Exenatide groups (all *p* < 0.001), consistent with substantial cardiomyocyte necrosis and membrane injury. Exenatide alone did not differ from the Control group for any biomarker (all *p* > 0.05). Importantly, Exenatide co-administration significantly attenuated the DOX-induced increases in cTnT (*p* < 0.001) (Figure 4A), CK (*p* < 0.01) (Figure 4B), CK-MB (*p* < 0.001) (Figure 4C), and LDH (*p* < 0.001) (Figure 4D). Nevertheless, biomarker levels in the Exenatide + DOX group remained modestly elevated compared with Control values (cTnT: *p* = 0.011; CK: *p* = 0.059; CK-MB: *p* = 0.041; LDH: *p* < 0.001), indicating partial but not complete biochemical normalization.

#### 3.2.2. Hepatocellular Injury and Renal Functional Markers

**Figure 5:**
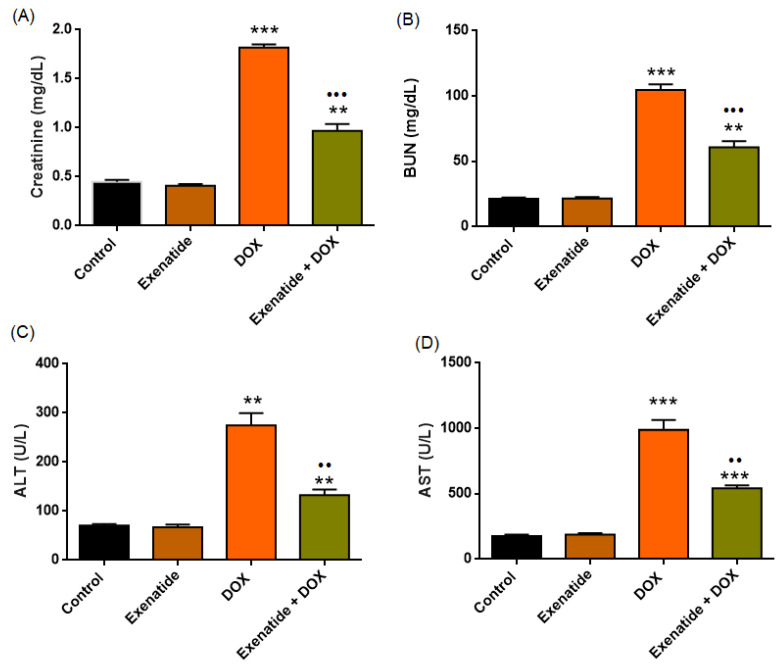
Effects of Exenatide on DOX-induced renal and hepatic injury biomarkers. (**A**) Serum creatinine (mg/dL), (**B**) blood urea nitrogen (BUN, mg/dL), (**C**) alanine aminotransferase (ALT, U/L), and (**D**) aspartate aminotransferase (AST, U/L) levels in Control, Exenatide, DOX, and Exenatide + DOX groups. DOX markedly elevated all renal (creatinine, BUN) and hepatic (ALT, AST) injury biomarkers, indicating substantial nephrotoxicity and hepatocellular damage. ** *p* < 0.01, *** *p* < 0.001 vs. Control; •• *p* < 0.01, ••• *p* < 0.001 vs. DOX.

Renal function analysis confirmed normal creatinine and BUN levels (Table 2). A highly significant treatment effect was observed for creatinine (F(3,24) = 247.026, *p* < 0.001) and BUN levels (F(3,24) = 141.253, *p* < 0.001). DOX administration increased creatinine and BUN when compared the control group (all *p* < 0.001). Exenatide alone showed no renal toxicity, with creatinine (*p* = 0.609) and BUN levels (*p* = 1.000) comparable to Control. Importantly, Exenatide co-treatment significantly attenuated the DOX-induced increases in both creatinine (*p* < 0.001) (Figure 5A) and BUN (*p* < 0.001) (Figure 5B), although values remained moderately elevated relative to Control (both *p* < 0.01) (Table 2).

Hepatic enzyme analysis results of all parameters are shown in Table 2. A highly significant treatment effect was observed for ALT (F(3,24) = 48.386, *p* < 0.001) and AST (F(3,24) = 93.787, *p* < 0.001). Across all enzymes, DOX markedly elevated hepatocellular injury markers compared with the Control group (ALT; *p* < 0.01; AST *p* < 0.001), confirming the presence of doxorubicin-induced hepatocellular injury. The difference in levels of AST and ALT between the Control and Exenatide groups was not statistically significant (*p* > 0.05). Importantly, Exenatide co-treatment significantly reduced DOX-induced increases in ALT and AST (both *p* < 0.01), although values remained modestly higher than in Controls (*p* < 0.01; *p* < 0.001, respectively) (Figure 5). Collectively, these results demonstrate that DOX induces substantial hepatocellular injury, as reflected by elevations across all major biochemical markers. In contrast, Exenatide confers broad but partial hepatoprotection, attenuating—but not completely normalizing—the DOX-mediated hepatic damage (Figure 5, Table 2).

### 3.3. SIRT-1 Analysis in Plasma (ng/mL) and Cardiac Tissue (ng/mg)

**Figure 6:**
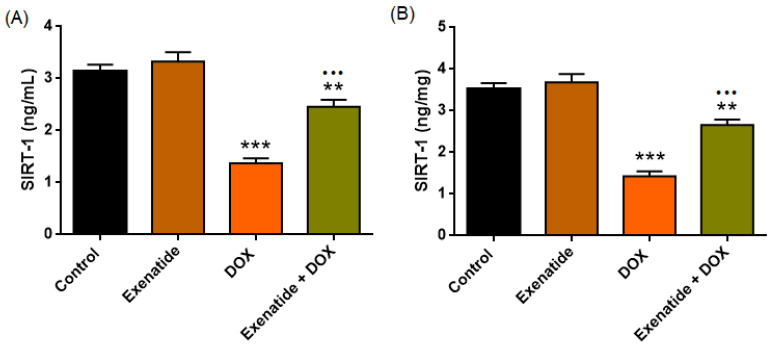
Plasma and myocardial SIRT-1 levels in experimental groups. (**A**) Plasma and (**B**) myocardial SIRT-1 concentrations in Control, Exenatide, DOX, and Exenatide + DOX groups. DOX markedly reduced SIRT-1 levels, whereas Exenatide co-treatment partially restored them. Data are mean ± SEM (*n* = 7). ** *p* < 0.01, *** *p* < 0.001 vs. Control; ••• *p* < 0.001, vs. DOX.

SIRT-1 analysis showed a highly significant treatment effect on plasma (F(3,24) = 42.191, *p* < 0.001) and tissue SIRT-1 levels (F(3,24) = 47.687, *p* < 0.001). DOX significantly reduced SIRT-1 levels in both plasma and myocardial tissue (*p* < 0.001), whereas the Exenatide + DOX group showed a marked increase in SIRT-1 compared with DOX alone (*p* < 0.001). Exenatide alone did not change its level when compared to control (Figure 6, Table 3). Also, Exenatide + DOX group showed significantly higher SIRT-1 levels than the Control group (both *p* < 0.01).

### 3.4. Oxidative Stress and Antioxidant Biomarkers

Oxidative stress markers showed significant group effects across all measured parameters (Nrf2: F(3,24) = 30.965, *p* < 0.001; MDA: F(3,24) = 70.312, *p* < 0.001; plasma GSH: F(3,24) = 35.092, *p* < 0.001; tissue GSH: F(3,24) = 40.163, *p* < 0.001; TAS: F(3,24) = 22.720, *p* < 0.001; TOS: F(3,24) = 60.391, *p* < 0.001).

**Figure 7:**
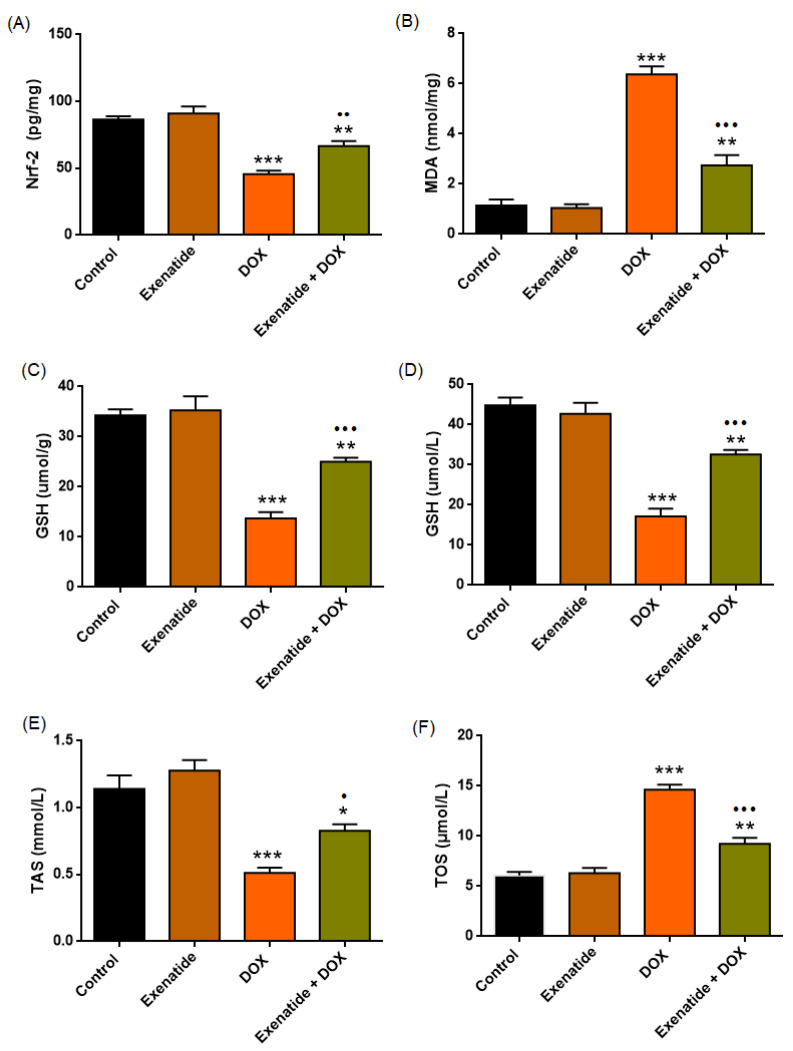
Effects of Exenatide on oxidative stress and antioxidant markers. (**A**) Myocardial Nrf2 (pg/mg), (**B**) MDA (nmol/mg), (**C**) Cardiac GSH (µmol/g) and (**D**) Plasma GSH (µmol/L). (**E**) Myocardial TAS (mmol/L) and (**F**) Myocardial TOS (µmol/L). * *p* < 0.05, ** *p* < 0.01, *** *p* < 0.001 vs. Control. • *p* < 0.05, •• *p* < 0.01, ••• *p* < 0.001 vs. DOX.

DOX administration markedly reduced myocardial Nrf2, GSH, and TAS, and increased myocardial MDA and TOS levels compared with Control (all *p* < 0.001). The Exenatide + DOX group showed a significant increase in Nrf2 (*p* < 0.01), GSH (plasma and myocardium) (*p* < 0.001), and TAS (*p* < 0.05) and reduced MDA (*p* < 0.001) and TOS (*p* < 0.001) levels (Figure 7, Table 3) when compared to the DOX group, but levels were still elevated relative to Control. Exenatide alone did not differ from oxidative parameters from Control (*p* > 0.05).

Values are presented as mean ± SEM (*n* = 7 per group). Statistical comparisons were performed using one-way ANOVA followed by the Tukey or Tamhane post hoc tests depending on variance homogeneity.

### 3.5. Inflammatory Biomarkers (NF-κB, TNF-α, IL-6) and NO

NF-κB p65 (ng/mg)

**Figure 8:**
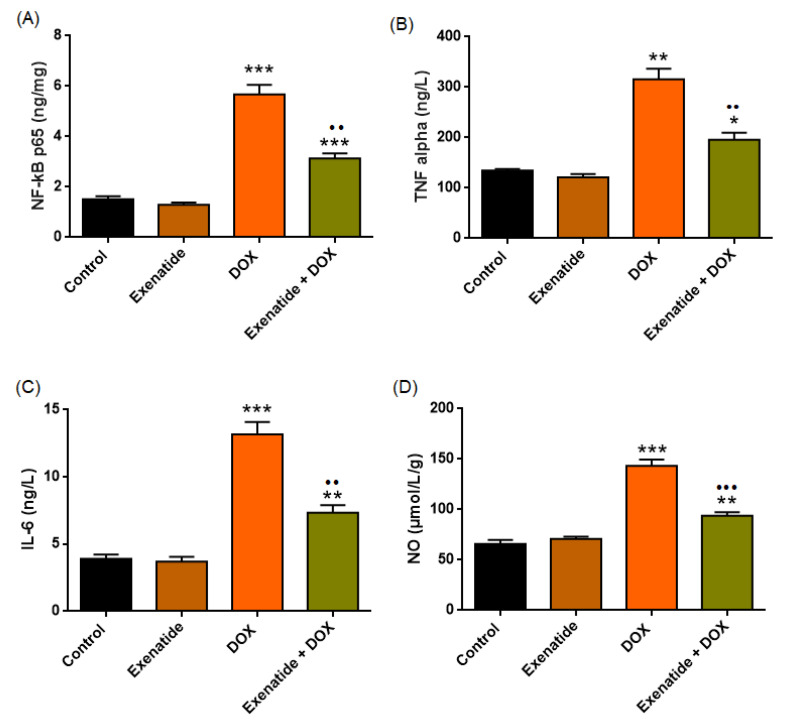
Inflammatory mediator levels in experimental groups. (**A**) NF-κB p65 (ng/mg), (**B**) TNF-α (ng/L), (**C**) IL-6 (ng/L), and (**D**) NO (µmol/L/g). DOX markedly increased all inflammatory markers, while Exenatide co-treatment significantly reduced these elevations. Data are mean ± SEM (*n* = 7). * *p* < 0.05, ** *p* < 0.01, *** *p* < 0.001 vs. Control. •• *p* < 0.01, ••• *p* < 0.001 vs. DOX.

NF-κB tissue analysis demonstrated a strong treatment effect on myocardial NF-κB expression (F(3,24) = 77.569, *p* < 0.001). DOX markedly increased NF-κB levels compared with both Control and Exenatide groups (all *p* < 0.001), indicating a pronounced pro-inflammatory response. Exenatide alone did not differ from Control (*p* = 0.638). Exenatide + DOX significantly reduced NF-κB activation relative to DOX (*p* < 0.01), although levels remained higher than in Control (*p* < 0.01) and Exenatide alone (*p* < 0.001) (Figure 8A, Table 4)

### 3.6. TNF-α and IL-6 Plasma

Pro-inflammatory cytokine analysis demonstrated a robust treatment effect on plasma TNF-α (F(3,24) = 44.987, *p* < 0.001) and IL-6 levels (F(3,24) = 56.664, *p* < 0.001). In both cytokines, doxorubicin administration caused a pronounced elevation compared with the Control group (*p* < 0.01; *p* < 0.001, respectively), indicating a strong systemic inflammatory response. Exenatide alone did not differ from Control (*p* > 0.05). Importantly, Exenatide co-administration significantly attenuated the doxorubicin-induced increases in TNF-α and IL-6 (both *p* < 0.01; Figure 8B,C). However, cytokine levels in the Exenatide + DOX group remained higher than those of the Control groups (TNF-α = *p* < 0.05; IL-6 = *p* < 0.01), suggesting that while Exenatide provides meaningful anti-inflammatory protection, it does not fully normalize the heightened cytokine response induced by doxorubicin (Table 4).

### 3.7. NO (µmol/L/g) (Heart Tissue)

Myocardial NO analysis revealed a strong treatment effect (F(3,24) = 68.162, *p* < 0.001). DOX markedly increased NO levels compared with Control (*p* < 0.001). Exenatide alone did not differ from Control (*p* = 0.841). Exenatide + DOX significantly reduced NO relative to DOX (*p* < 0.001), yet values remained higher than in Control (*p* = 0.01) (Figure 8D, Table 4).

### 3.8. 99mTc-PYP Uptake

**Figure 9:**
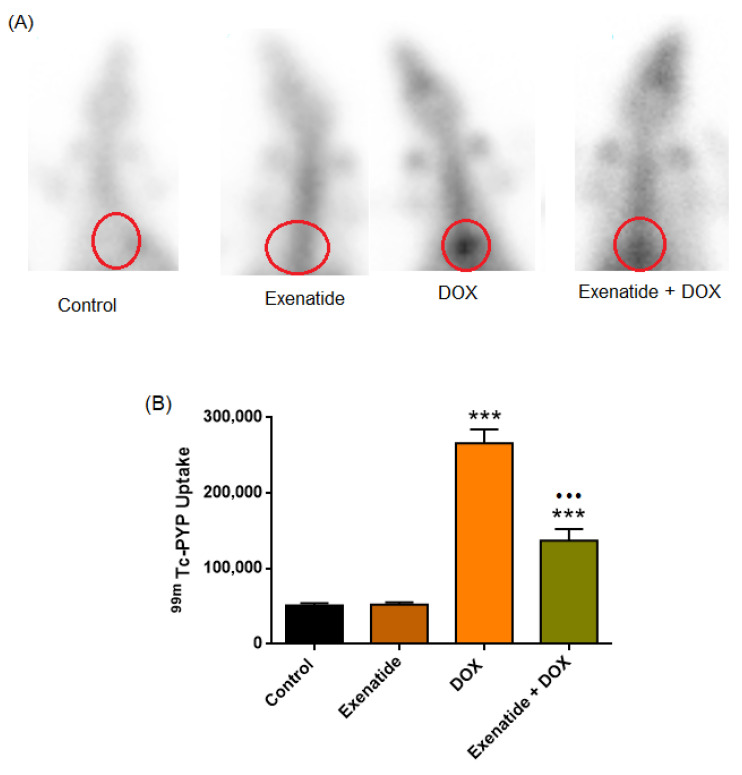
Representative 99mTc-PYP cardiac scintigraphy images and quantitative uptake values in the four experimental groups. Upper panel (**A**): planar anterior 99mTc-PYP images of Control, Exenatide, DOX, and Exenatide + DOX groups, with the myocardial region of interest indicated by red circles. Lower left panel: whole-body 99mTc-PYP projections illustrating the visually increased cardiac tracer uptake in the DOX group and its partial attenuation in the Exenatide + DOX group. Lower right panel (**B**): bar graph showing 99mTc-PYP uptake (mean ± SEM); DOX markedly increased myocardial tracer accumulation compared with Control (*** *p* < 0.001), whereas Exenatide + DOX significantly reduced DOX-induced uptake (••• *p* < 0.001).

Normality assessment (Shapiro–Wilk) indicated deviation from normality in at least one group (DOX, *p* = 0.024). Therefore, overall group differences were evaluated using the Kruskal–Wallis test, followed by Mann–Whitney U tests for predefined pairwise comparisons with appropriate multiple-comparison adjustment. No significant difference was observed between the Control and Exenatide groups (51,012 ± 3153 vs. 52,370 ± 2631; *p* > 0.05). In contrast, DOX treatment resulted in a marked increase in myocardial 99mTc-PYP uptake compared with Control (266,190 ± 18,305; *p* < 0.001). Exenatide co-administration significantly attenuated DOX-induced tracer uptake when compared with the DOX group (*p* < 0.001). However, uptake values in the Exenatide + DOX group remained significantly higher than those of the Control group (136,821 ± 15,659; *p* < 0.001). (Figure 9).

## 4. Discussion

In the present study, the cardioprotective effects of Exenatide against DOX-induced cardiotoxicity were comprehensively evaluated through an integrated experimental platform combining functional assessments, in vivo 99mTc-PYP scintigraphy, and molecular signaling analyses. In this context, 99mTc-PYP imaging provided in vivo evidence of reduced myocardial injury burden in Exenatide-treated animals, complementing the parallel improvements observed in electrocardiographic indices. At the mechanistic level, our data are consistent with a SIRT-1-linked pathway, in which enhanced Nrf2-dependent antioxidant responses and attenuation of NF-κB-associated inflammatory signaling accompany the reduction in injury. The concordance between imaging, functional, and molecular endpoints strengthens the translational relevance of Exenatide in DOX-induced cardiotoxicity. It suggests that SIRT-1-centered redox–inflammatory modulation may represent a plausible mechanistic axis contributing to this protective effect.

In our study, DOX markedly increased cardiac 99mTc-PYP uptake. Assessment of DOX-induced cardiac injury is enhanced by technetium-99m pyrophosphate (99mTc-PYP) scintigraphy, which binds calcium deposits in necrotic cardiomyocytes [14,15]. Elevated myocardial uptake has been reported in various forms of myocardial injury, particularly in anthracycline-induced cardiotoxicity [11,16,17]. During ischemic damage, intracellular Ca^2+^ accumulates in mitochondria as calcium-phosphate complexes, to which 99mTc-PYP exhibits high affinity [18]. Owing to its stability and rapid clearance, Tc-99m PYP serves as a reliable marker of acute, irreversible myocardial injury, with tracer uptake diminishing as necrotic tissue is cleared by phagocytosis [16,19,20]. In this context, the markedly lower 99mTc-PYP uptake in the Exenatide-treated group indicates a substantial mitigation of DOX-induced myocardial injury. Furthermore, the concordance of PYP scintigraphy findings with clinically used serum cardiac biomarkers and ECG-derived measures suggests that PYP scintigraphy may enhance the translational relevance of experimental cardiotoxicity assessment.

In this study, ECG after DOX administration showed significant changes. Consistent with earlier reports, DOX treatment reduces heart rate, prolongs the QT interval, and elevates the ST segment, which may be linked to its deleterious effects on the cell membrane [21,22]. QT prolongation and ST elevation are consistently reported. They are considered hallmark electrophysiological alterations linked to DOX-induced oxidative stress, impaired repolarization (particularly via IKs_ss inhibition), calcium overload, and early myocardial membrane injury [23,24,25,26]. Exenatide treatment attenuated DOX-induced ECG changes, likely through its membrane-stabilizing effect.

In the present study, DOX cardiotoxicity manifested as significant elevations in serum LDH, CK, CK-MB, and cTnT, accompanied by increased cardiac oxidative stress markers (NO, MDA, TOS) and reductions in antioxidant defenses (Nrf2, GSH, TAS). Serum troponin-T, CK, CK-MB, and LDH are widely recognized as sensitive biochemical markers of cardiotoxicity and myocardial dysfunction, and numerous studies have consistently reported marked elevations during cardiotoxic insults [27]. Earlier studies have shown that DOX promotes lipid peroxidation, destabilizes cellular membranes, and facilitates cytosolic enzyme leakage, clarifying key mechanisms underlying its cardiotoxicity [28,29]. Ca^2+^ overload may further impair membrane structure by activating phospholipases [30]. The parallel rise in MDA and TOS suggests excessive ROS generation overwhelming the heart’s antioxidant defenses, as evidenced by depleted GSH and TAS levels [31,32,33,34]. DOX-related inhibition of Nrf2 signaling likely weakens transcriptional antioxidant responses, exacerbating ROS accumulation. Previous studies have shown that Exenatide reduces myocardial MDA, enhances antioxidant capacity, and suppresses lipid peroxidation in cardiotoxicity models through GLP-1R-mediated activation of the Nrf2 pathway [10,35]. Exenatide increased Nrf2 and GSH and lowered MDA, TOS, and serum injury markers, suggesting that its cardioprotection is likely mediated by Nrf2-dependent redox restoration.

In this study, DOX exposure led to significant NF-κB activation and NO elevation, consistent with previous reports [22,36,37]. DOX-induced cardiotoxicity is strongly associated with oxidative stress and NF-κB-mediated inflammation, both of which contribute to myocardial injury [38,39,40]. NF-κB activation amplifies pro-inflammatory cytokines such as TNF-α and IL-6, and upregulates iNOS, promoting excessive cardiac NO production, a hallmark of early damage [41,42]. NF-κB-induced NO elevation may help explain the DOX-induced bradycardia observed in this model.

SIRT1, a NAD^+^-dependent deacetylase, negatively regulates NF-κB and supports redox balance via Nrf2 signaling [43]. Numerous experimental studies have demonstrated that reduced SIRT1 expression—or even genetic deletion of SIRT-1—significantly exacerbates DOX-induced cardiac injury, by reducing oxidative stress and inflammation through Nrf2 activation and NF-κB suppression [4,44,45,46,47]. Exenatide treatment reversed these effects by restoring SIRT1/Nrf2 signaling and suppressing NF-κB, thereby reducing inflammation and oxidative stress. These findings align with earlier data showing GLP-1 agonists protect the heart through SIRT1-mediated anti-inflammatory and antioxidant mechanisms [10,48]. The present study suggests that Exenatide mitigates DOX-induced cardiotoxicity primarily by restoring SIRT-1/Nrf2-mediated antioxidant defenses and suppressing NF-κB-driven inflammatory signaling, thereby preserving myocardial structural and functional integrity.

Notably, concurrent reductions in SIRT-1 levels in both cardiac tissue and circulation were observed alongside electrocardiographic abnormalities, increased myocardial 99mTc-PYP uptake, and elevated serum cardiac injury markers. These findings suggest that SIRT-1 may reflect multiple dimensions of DOX-induced cardiac injury and may serve as a potential indicator of cardiotoxicity. However, further studies are required to clarify causality and clinical relevance.

We demonstrated that DOX administration resulted in marked increases in serum AST and ALT levels, together with elevated BUN and creatinine concentrations [49,50]. These findings clearly indicate DOX-induced hepatocellular and renal injury, consistent with systemic oxidative stress and inflammatory damage driven by excessive ROS generation and lipid peroxidation [51]. Notably, Exenatide co-treatment significantly attenuated these DOX-induced elevations, suggesting a partial protection against organ toxicity, as previously reported [35]. This protective effect is most likely mediated by Exenatide’s antioxidant and anti-inflammatory properties, which help preserve cellular membrane integrity and limit the leakage of injury-related biomarkers into the circulation.

### Limitations

In the present study, histological analyses of cardiac tissue, including hematoxylin–eosin staining, were not performed. This represents a limitation in demonstrating myocardial injury at the structural level. Future studies incorporating histopathological evaluations may provide more detailed morphological support for the scintigraphic findings.

Mechanistic overview of doxorubicin-induced cardiotoxicity and the protective effects of Exenatide: Doxorubicin (DOX) administration promotes excessive reactive oxygen species (ROS) production, leading to oxidative stress, lipid peroxidation, endothelial dysfunction, and increased vascular permeability. These processes are associated with elevated circulating cardiac (CK, CK-MB, cTnT, LDH), hepatic (AST, ALT), renal (BUN, creatinine), and inflammatory (TNF-α, IL-6) biomarkers. At the cellular level, DOX-induced ROS overproduction suppresses antioxidant defenses (GSH, TAS). It activates pro-inflammatory signaling pathways, including NF-κB and iNOS-derived nitric oxide (NO), ultimately resulting in myocardial damage. Exenatide exerts cardioprotective effects by activating the GLP-1 receptor, enhancing SIRT1 signaling, and promoting nuclear translocation of Nrf2, thereby reducing oxidative stress markers (MDA, TOS), restoring antioxidant capacity, attenuating inflammatory signaling, and limiting myocardial injury.

## Figures and Tables

**Table 1:** ECG findings in the 4 groups of 7 rats.

	Control	Exenatide	DOX	Exenatide + DOX
Heart rate (bpm)	302 ± 10	319 ± 12	236 ± 11 ^b^	276 ± 5 ^c^
QT interval (duration, s)	0.0700 ± 0.003	0.068 ± 0.003	0.098 ± 0.002 ^b^	0.082 ± 0.001 ^a,d^
ST-segment amplitude (mV)	0.058 ± 0.004	0.054 ± 0.004	0.12 ± 0.008 ^b^	0.095 ± 0.004 ^b,d^

Values expressed the mean ± SEM; ^a^ = *p* < 0.05, ^b^ = *p* < 0.001 vs. Control; *p* < 0.05 vs. Control; ^c^ = *p* < 0.05, ^d^ = *p* < 0.01 vs. DOX.

**Table 2:** Mean serum enzyme levels in the 3 groups of rats.

	Control	Exenatide	DOX	Exenatide + DOX
cTnT (pg/mL)	1229 ± 153	1182 ± 122	3073 ± 198 ^c^	1988 ± 138 ^a,f^
CK (U/L)	331 ± 27	324 ± 15	613 ± 36 ^c^	435 ± 26 ^e^
CK-MB (U/L)	437 ± 40	425 ± 31	847 ± 43 ^c^	590 ± 35 ^a,f^
Creatinine (mg/dL)	0.45 ± 0.01	0.40 ± 0.01	1.81 ± 0.03 ^c^	0.96 ± 0.07 ^b,f^
BUN (mg/dL)	21.41 ± 0.89	22.01 ± 1.16	104.7 ± 4.46 ^c^	60.83 ± 4.72 ^b,f^
ALT (U/L)	70.71 ± 2.7	67.14 ± 5.2	274 ± 24 ^b^	132 ± 11 ^b,e^
AST (U/L)	177 ± 11	188 ± 10	988 ± 73 ^c^	541 ± 23 ^c,e^
LDH (U/L)	419 ± 23	388 ± 22	1227 ± 47 ^c^	746 ± 30 ^c,f^

Values expressed the mean ± SEM. ^a^ = *p* < 0.05, ^b^ = *p* < 0.01, ^c^ = *p* < 0.001 vs. Control, ^e^ = *p* < 0.01, ^f^ = *p* < 0.001 vs. DOX.

**Table 3:** Mean oxidative stress and antioxidant markers in the 4 groups of rats.

	Control	Exenatide	DOX	Exenatide + DOX
SIRT-1 (ng/mL)	3.14 ± 0.12	3.32 ± 0.17	1.37 ± 0.09 ^c^	2.45 ± 0.13 ^b,f^
SIRT-1 (ng/mg)	3.52 ± 0.13	3.68 ± 0.19	1.42 ± 0.12 ^c^	2.65 ± 0.14 ^b,f^
Nrf2 (pg/mg)	86 ± 2.4	91 ± 5.3	45 ± 2.6 ^c^	66 ± 3.7 ^b,e^
MDA (nmol/mL)	1.14 ± 0.24	1.04 ± 0.15	6.37 ± 0.31 ^c^	2.74 ± 0.41 ^b,f^
GSH (umol/g)	34.29 ± 1.14	35.29 ± 2.80	13.71 ± 1.26 ^c^	25 ± 0.81 ^b,f^
GSH (umol/L)	44.86 ± 1.90	42.71 ± 2.71	17.14 ± 1.93 ^c^	32.57 ± 1.09 ^b,f^
TAS (mmol/L)	1.14 ± 0.10	1.27 ± 0.07	0.51 ± 0.03 ^c^	0.82 ± 0.050 ^a,d^
TOS (µmol/L)	6.09 ± 0.35	6.30 ± 0.52	14.62 ± 0.53 ^c^	9.24 ± 0.60 ^b,f^

^a^ = *p* < 0.05, ^b^ = *p* < 0.01, ^c^ = *p* < 0.001 vs. Control, ^d^ = *p* < 0.05, ^e^ = *p* < 0.01, ^f^ = *p* < 0.001 vs. DOX.

**Table 4:** Mean inflammatory markers in the 4 groups of rats.

	Control	Exenatide	DOX	Exenatide + DOX
NF-kB p65 (ng/mg)	1.51 ± 0.11	1.30 ± 0.08	5.67 ± 0.38 ^c^	3.13 ± 0.19 ^c,d^
TNF alpha (ng/L)	134 ± 2.8	121 ± 6	315 ± 21 ^b^	195 ± 14 ^a,d^
IL-6 (ng/L)	3.90 ± 0.31	3.71 ± 0.35	13.17 ± 0.91 ^c^	7.34 ± 0.56 ^b,d^
NO (µmol/L/g)	65 ± 4	70 ± 2	143 ± 6 ^c^	93 ± 3 ^b,e^

^a^ = *p* < 0.05, ^b^ = *p* < 0.01, ^c^ = *p* < 0.001 vs. Control, ^d^ = *p* < 0.01, ^e^ = *p* < 0.001 vs. DOX. Values are presented as mean ± SEM (*n* = 7 per group).

## Data Availability

The data that support the findings of this study are not publicly available due to ethical reasons but are available from the corresponding author upon request.
